# Fatty acid profiles of muscle, liver, heart and kidney of Australian prime lambs fed different polyunsaturated fatty acids enriched pellets in a feedlot system

**DOI:** 10.1038/s41598-018-37956-y

**Published:** 2019-02-04

**Authors:** Hung Van Le, Don Viet Nguyen, Quang Vu Nguyen, Bunmi Sherifat Malau-Aduli, Peter David Nichols, Aduli Enoch Othniel Malau-Aduli

**Affiliations:** 1 Animal Genetics and Nutrition, Veterinary Sciences Discipline, College of Public Health, Medical and Veterinary Sciences, Division of Tropical Health and Medicine, James Cook University, Townsville, Queensland 4811 Australia; 2 National Institute of Animal Science, Thuy Phuong, Bac Tu Liem, Hanoi 129909 Vietnam; 3 College of Economics and Techniques, Thai Nguyen University, Thai Nguyen, 252166 Vietnam; 4 College of Medicine and Dentistry, Division of Tropical Health and Medicine, James Cook University, Townsville, Queensland 4811 Australia; 5 CSIRO Oceans & Atmosphere, PO Box 1538, Hobart, TAS 7001 Australia

## Abstract

We investigated the effect of various dietary polyunsaturated fatty acid (PUFA) sources on the fatty acid profiles of muscle, liver, heart and kidney of Australian prime lambs. Seventy-two White Suffolk x Corriedale first-cross lambs weaned at 6 months of age were randomly allocated to the following six treatments: (1) Control: Lucerne hay only; wheat-based pellets infused with 50 ml/kg dry matter (DM) of oil from (2) rice bran (RBO); (3) canola (CO); (4) rumen-protected (RPO), (5) flaxseed (FSO) and (6) safflower (SO) sources in a completely randomized experimental design. Lambs in CO, FSO, SO and RPO treatments achieved contents of eicosapentaenoic acid (EPA, 22:5n-3) plus docosahexaenoic acid (DHA, 22:6n-3) in the *longissimus dorsi* muscle ranging from 31.1 to 57.1 mg/135 g, over and above the 30 mg per standard serve (135 g) threshold for “source” claim under the Australian guidelines. There was no difference in n-3 LC-PUFA contents in *longissimus dorsi* muscle of lambs fed dietary oils of plant origin. The highest 18:3n-3 (ALA) contents achieved with FSO diet in the muscle, liver and heart were 45.6, 128.1 and 51.3 mg/100 g, respectively. Liver and kidney contained high contents of n-3 LC-PUFA (ranging from 306.7 to 598.2 mg/100 g and 134.0 to 300.4 mg/100 g, respectively), with all values readily exceeding the ‘good source’ status (60 mg per serve under Australian guidelines). The liver and kidney of PUFA fed lambs can be labelled as ‘good source’ of n-3 LC-PUFA based on EPA and DHA contents stipulated by the Food Standards of Australia and New Zealand guidelines. Therefore, if lamb consumers consider eating the liver and kidney as their dietary protein sources, they can adequately obtain the associated health benefits of n-3 LC-PUFA.

## Introduction

Accumulating scientific evidence demonstrates the important role that omega-3 long-chain (>C_20_) polyunsaturated fatty acids (n-3 LC-PUFA) play in conditions such as cardiovascular disease, certain cancers and other diseases^1,2^. Chen *et al*.^3^ suggested that both dietary and circulating LC-PUFA were inversely associated with all-cause mortality. In another study, Stark *et al*.^4^ found that most of the countries and regions of the world having very low to low range of blood n-3 LC-PUFA were associated with an increased risk of chronic diseases. Therefore, the consumption of adequate n-3 LC-PUFA is crucial to maintaining a healthy body and for the prevention of chronic diseases. However, humans like all other mammals, cannot synthesise n-3 LC-PUFA because they lack the necessary Δ12 and Δ15-desaturase enzymes. Therefore, these health-benefitting fatty acids must be supplied in the diet^5^. Humans can obtain n-3 LC-PUFA or their C_18_ PUFA precursors from various sources including aquatic (fish, crustaceans, and mollusks), farm livestock products (meat, egg, and milk), oilseeds, fruits, herbs, cyanobacteria and microorganisms (bacteria, fungi, microalgae, and diatoms)^6^. Ruminant meat research has drawn considerable attention because ruminant meat contains some bioactive lipids (including n-3 LC-PUFA) and the fatty acid profiles of ruminant meat can be enhanced through dietary supplementation^7,8^. In this regard, the manipulation of fatty acid profiles in sheep meat via dietary n-3 LC-PUFA supplementation has yielded some promising results. Flakemore *et al*.^9^ reported that supplementation of degummed crude canola oil to lambs improved n-3 LC-PUFA contents in meat. Supplementing prime lambs with 5% flaxseed or canola oil pellets increased n-3 LC-PUFA in the liver, heart, kidney and *longissimus thoracis et lumborum* muscle tissue^10,11^. Boles *et al*.^12^ found that supplementation of safflower oil at up to 6% of sheep diet on as-fed basis, increased levels of PUFA and conjugated linoleic acids (especially the cis-9, trans-11 isomer) in the lean tissue. However, the studies described above emphasised the need to find new n-3 LC-PUFA feed sources and associated optimal supplementation levels. There are only a handful of published comparative studies into different dietary oil sources and their ability to elevate the level of n-3 LC-PUFA in lamb, especially under feedlot finishing systems. Feeding lambs in an intensive finishing system, such as a feedlot, has rapidly become a specialised component of the prime lamb industry with the number of lambs being grain finished steadily increasing. This increase in intensive feeding can be mainly attributed to the export demand for a consistent supply of lambs that meet market specifications. This is particularly so when quality pasture feed is unavailable or during drought conditions. Therefore, we hypothesised herein that the fatty acid profiles of the *longissimus dorsi* muscle, liver, kidney and heart of Australian prime lambs would differ in response to supplementation with various dietary PUFA sources under a typical intensive, in-door feedlot management system. It was intended that this study would provide outcomes that can be used as guidelines for lamb producers for a better utilisation of available dietary n-3 LC-PUFA sources for enhancing lamb nutritional quality and beneficial human health outcomes.

## Results

### Fatty acid profiles in basal and supplementary feed

Fatty acid composition (as % total fatty acids) of both basal and supplementary feeds are presented in Table 1. Lucerne hay contained high proportions of PUFA and n-3 PUFA (48.7 and 28.2%, respectively). The major fatty acids in lucerne hay were 18:2n-6 (LA) and 18:3n-3 (ALA), with relatively high levels of 19.0 and 27.5%, respectively. Similarly, flaxseed oil infused pellets (FSO) contained relatively high levels of PUFA and n-3 PUFA (62.2 and 20.7%, respectively) and the major fatty acids in FSO were LA (41.0%), ALA (20.5%) and 18:1n-9 (18.8%). The remaining treatments were rich in PUFA and n-6 PUFA with relatively high levels ranging from 39 to 59 (%) and 34 to 57(%), respectively, in which the major fatty acids were LA and 18:1n-9. The highest n-6 PUFA level was found in safflower oil infused pellets (SO) (mainly LA: 56.5%). The rumen protected oil infused pellets (RPO) had the highest proportions of n-3 LC-PUFA comprising 4.8% eicosapentaenoic acid (EPA, 20:5n-3), docosapentaenoic acid (DPA, 22:5n-3) and docosahexaenoic acid (DHA, 22:6n-3).

**Table 1:** Fatty acid composition (% total fatty acids) of basal and supplementary feeds.

Fatty acid	Lucerne hay	RBO	CO	FSO	SO	RPO
14:0	0.4	0.1	0.1	0.1	0.2	2.4
15:0	0.5	0.1	0.1	0.1	0.1	0.4
16:1n-9c	0.0	0.0	0.0	0.0	0.0	0.0
16:1n-7c	0.5	0.2	0.3	0.3	0.2	4.0
16:0	26.6	17.4	12.4	11.0	14.0	19.4
17:0	0.7	0.1	0.1	0.1	0.1	0.2
18:2n-6 LA	19.0	41.1	33.7	41.0	56.5	35.7
18:3n-3 ALA	27.5	2.3	4.8	20.5	1.7	2.7
18:1n-9	2.7	31.8	41.8	18.8	20.3	20.9
18:1n-7c	1.0	1.1	2.5	1.8	1.5	1.9
18:1n-7t	0.1	0.0	0.0	0.0	0.0	0.0
18:0	5.5	2.3	1.1	3.4	2.3	2.9
20:4n-6 ARA	0.2	0.0	0.0	0.0	0.0	0.2
20:5n-3 EPA	0.2	0.1	0.1	0.1	0.1	2.5
20:3n-6	0.4	0.2	0.1	0.3	0.5	0.4
20:4n-3	0.5	0.0	0.0	0.0	0.0	0.1
20:2n-6	0.3	0.1	0.1	0.1	0.1	0.1
20:0	1.6	0.6	0.6	0.4	0.5	0.4
22:5n-6 DPA-6	0.0	0.0	0.0	0.0	0.0	0.2
22:6n-3 DHA	0.0	0.0	0.0	0.0	0.0	1.6
22:5n-3 DPA-3	0.0	0.0	0.0	0.0	0.0	0.5
22:0	2.0	0.5	0.3	0.3	0.2	0.4
23:0	1.0	0.1	0.1	0.1	0.1	0.1
24:0	2.3	0.6	0.4	0.2	0.3	0.4
∑SFA	40.9	21.9	15.1	16.0	17.9	26.7
∑MUFA	9.7	34.0	45.7	21.8	22.9	28.3
∑PUFA	48.7	43.9	39.0	62.2	59.0	44.6
∑n-3 LC-PUFA	0.7	0.1	0.1	0.1	0.1	4.8
∑n-3 PUFA	28.2	2.4	4.9	20.7	1.7	7.4
∑n-6 PUFA	20.2	41.4	34.0	41.4	57.0	37.1
∑other FA	0.8	0.2	0.2	0.1	0.2	0.4
n-6/n-3	0.7	17.3	6.9	2.0	33.5	5.0

RBO, CO, RPO, FSO and SO were wheat-based pellets infused with 50 ml/kg DM of oil from rice bran, canola, rumen-protected, flaxseed and safflower sources, respectively; LA, linoleic acid; ALA, α-linolenic acid; EPA, eicosapentaenoic acid; DHA, docosahexaenoic acid; DPA, docosapentaenoic acid; ΣSFA, total saturated fatty acids; ΣMUFA, total monounsaturated fatty acids; and total polyunsaturated fatty acids (ΣPUFA);

∑SFA is the sum of 14:0, 15:0, 16:0, 17:0, 18:0, 20:0, 21:0, 22:0, 23:0, 24:0;

∑MUFA is the sum of 14:1, 16:1n-13t, 16:1n-9, 16:1n-7, 16:1n-7t, 16:1n-5c, 17:1n-8 + a17:0, 18:1n-9, 18:1n-7t, 18:1n-5, 18:1n-7, 18:1a, 18:1b, 18:1c, 19:1a, 19:1b, 20:1n-11, 20:1n-9, 20:1n-7, 20:1n-5, 22:1n-9, 22:1n-11, 24:1n-9;

∑PUFA is the sum of 18:4n-3, 18:3n-6, 18:2n-6, 18:3n-3, 20:3, 20:4n-3, 20:4n-6, 20:5n-3, 20:3n-6, 20:2n-6, 22:6n-3, 22:5n-3, 22:5n-6, 22:4n-6;

∑n-3 LC-PUFA is the sum of 20:5n-3, 20:4n-3, 22:6n-3, 22:5n-3;

∑n-3 PUFA is the sum of 18:3n-3, 18:4n-3, 20:4n-3, 20:5n-3, 22:6n-3, 22:5n-3;

∑n-6 PUFA is the sum of 18:2n-6, 18:3n-6, 20:4n-6, 20:3n-6, 20:2n-6, 22:5n-6, 22:4n-6;

∑other FA is the sum of other individual FA present at <0.1% except ARA, DHA, EPA, and DPA.

### Fatty acid profiles in the longissimus dorsi muscle

Fatty acid contents (mg/100 g) of the *longissimus dorsi* muscle tissue is depicted in Table 2. There was no difference among treatments in total fatty acids (FA), saturated fatty acids (SFA) and monounsaturated fatty acids (MUFA). The PUFA, n-3 LC-PUFA, n-3 PUFA and 18:1n-7t of supplemented treatments were significantly higher than that of control. The RPO treatment had the highest contents of n-3 LC-PUFA (66 mg/100 g); mainly EPA, DPA and DHA. The highest mean of ALA was found in the FSO treatment (45.6 mg/100 g) and the lowest value was in the control. All supplemented treatments, except for RPO, had significantly higher values of LA than that of control. The SO treatment contained the highest contents of n-6 PUFA and n-6/n-3 ratios of all treatments varied from 1.8 to 3.5.

**Table 2:** Fatty acid contents (mg/100 g) of *longissimus dorsi* muscle tissue in lambs fed various dietary PUFA enriched pellets.

Fatty acid	Control	RBO	CO	FSO	SO	RPO	SEM	P
14:0	24.1^ab^	34.6^a^	30.8^ab^	23.5^ab^	26.3^ab^	18.4^b^	4.81	0.047
15:0	4.6	5.9	4.9	3.5	4.2	3.5	0.77	0.259
16:1n-9c	3.1^ab^	4.0^a^	4.0^a^	2.6^ab^	3.1^ab^	2.4^b^	0.49	0.041
16:1n-7c	16.6	23.1	21.0	16.8	18.8	16.3	2.64	0.380
16:0	375.7	498.8	484.9	386.2	460.7	342.0	55.69	0.266
17:0	20.6	25.1	20.3	16.8	21.0	17.2	3.04	0.444
18:2n-6 LA	87.0^c^	158.5^ab^	140.0^b^	148.7^b^	196.8^a^	122.9^bc^	14.58	0.000
18:3n-3 ALA	23.9^c^	31.2^bc^	35.8^b^	45.6^a^	29.5^bc^	25.6^c^	2.95	0.000
18:1n-9	601.6	794.6	776.2	558.3	720.9	538.6	92.86	0.227
18:1n-7c	23.8^b^	36.6^a^	40.0^a^	31.1^ab^	36.1^a^	31.6^ab^	3.73	0.009
18:1n-7t	22.1^b^	50.3^a^	47.6^a^	50.4^a^	48.4^a^	42.2^a^	6.68	0.041
18:0	323.5	386.4	387.3	299.6	382.5	247.5	47.89	0.236
20:4n-6 ARA	27.9^b^	36.1^ab^	33.7^ab^	30.1^b^	45.2^a^	29.1^b^	4.45	0.046
20:5n-3 EPA	12.6^c^	16.0^bc^	18.1^bc^	19.8^b^	18.1^bc^	28.9^a^	2.02	0.000
20:3n-6	5.9^ab^	3.9^b^	5.6^ab^	5.3^ab^	6.7^a^	6.9^a^	0.74	0.048
20:4n-3	1.9^bc^	2.8^ab^	1.2^bc^	1.1^bc^	1.0^c^	4.2^a^	0.55	0.002
20:2n-6	1.4	1.6	1.7	1.4	2.2	2.3	0.30	0.168
20:0	2.4	3.1	3.0	2.2	2.9	2.2	0.39	0.383
22:5n-6 DPA-6	0.6^ab^	0.7^a^	0.5^ab^	0.3^b^	0.6^ab^	0.9^a^	0.13	0.042
22:6n-3 DHA	3.7^b^	4.3^b^	4.9^b^	5.4^b^	5.4^b^	13.3^a^	0.95	<0.0001
22:5n-3 DPA-3	10.1^b^	15.2^ab^	16.8^a^	14.4^ab^	18.0^a^	19.6^a^	1.97	0.033
22:0	1.2	1.3	1.2	1.1	1.2	0.9	0.17	0.605
23:0	1.9	1.5	1.4	1.3	1.5	1.3	0.23	0.462
24:0	1.9	1.8	1.6	1.3	1.7	1.8	0.22	0.534
Total FA	1748.0	2297.0	2241.0	1821.0	2218.0	1658.0	239.60	0.247
∑SFA	755.9	958.4	935.5	735.5	902.0	634.9	110.76	0.259
∑MUFA	717.0	966.8	947.5	713.3	883.2	677.1	109.81	0.254
∑PUFA	183.4^b^	279.2^a^	266.1^a^	279.7^a^	332.9^a^	260.8^a^	25.55	0.012
∑n-3 LC-PUFA	28.4^c^	38.3^b^	41.0^b^	40.7^b^	42.5^b^	66.0^a^	4.51	<0.0001
∑n-3 PUFA	52.2^d^	69.6^c^	76.7^abc^	86.9^ab^	72.7^bc^	91.6^a^	6.55	0.003
∑n-6 PUFA	124.8^c^	204.2^ab^	183.9^bc^	188.1^b^	254.9^a^	164.3^bc^	19.71	0.003
∑other FA	91.7	92.6	91.9	92.1	100.1	84.9	8.75	0.908
n-6/n-3	2.4^c^	3.0^b^	2.4^c^	2.2^cd^	3.5^a^	1.8^d^	0.16	<0.0001

^*^Values within the same row bearing different superscripts differ (P < 0.05). Total FA is the combined FA contents. All other abbreviations are as defined in Table 1.

### Fatty acid profiles in edible organs

#### Fatty acid profiles in the liver

Table 3 shows the fatty acid contents (mg/100 g) of liver tissue. The means of all fatty acid components in the liver were markedly higher than those in the muscle and significant differences were evident in both saturated and unsaturated FA. The CO treatment had the highest total FA (3775.0 mg/100 g) and MUFA (1018.0 mg/100 g) contents. The lowest SFA and 18:0 contents were found in the RPO group (1086.0 mg/100 g and 595.5 mg/100 g, respectively). Contents of n-6 PUFA and LA in all supplemented treatments, except for RPO, were significantly higher than in the control. RPO had the highest n-3 PUFA and n-3 LC-PUFA which mainly consisted of EPA, DPA and DHA. The ratio of n-6/n-3 in all treatments ranged from 0.6 to 1.9. The highest value of ALA was found in the FSO treatment (128.1 mg/100 g).

**Table 3:** Fatty acid contents (mg/100 g) of the liver.

Fatty acid	Control	RBO	CO	FSO	SO	RPO	SEM	P
14:0	10.0^a^	10.0^a^	10.2^a^	8.5^ab^	7.7^ab^	4.7^b^	1.43	0.043
15:0	12.0^a^	7.3^bc^	9.0^b^	6.9^bc^	6.4^bc^	5.6^c^	0.86	0.000
16:1n-9c	10.5^ab^	11.1^ab^	12.9^a^	8.0^bc^	9.2^ab^	4.9^c^	1.31	0.004
16:1n-7c	20.4^a^	18.1^ab^	17.9^ab^	11.7^c^	12.8^bc^	13.4^bc^	1.94	0.017
16:0	526.9^a^	520.5^a^	541.8^a^	446.9^ab^	453.0^ab^	396.0^b^	35.51	0.044
17:0	56.9^a^	39.2^b^	44.7^b^	42.6^b^	41.5^b^	42.9^b^	2.61	0.001
18:2n-6 LA	209.5^b^	407.0^a^	389.3^a^	411.1^a^	434.5^a^	227.4^b^	30.30	<0.0001
18:3n-3 ALA	61.6^c^	70.2^bc^	104.3^ab^	128.1^a^	72.5^bc^	48.5^c^	11.83	0.000
18:1n-9	566.8^ab^	585.4^ab^	668.0^a^	423.6^cd^	459.1^bc^	313.1^d^	46.77	0.000
18:1n-7c	36.8^b^	48.1^ab^	52.4^ab^	41.3^b^	93.5^a^	64.3^ab^	15.95	0.041
18:1n-7t	57.1^b^	118.8^ab^	147.8^a^	172.9^a^	115.0^ab^	146.5^a^	22.77	0.023
18:0	748.8^a^	718.2^a^	792.8^a^	743.0^a^	737.8^a^	595.5^b^	30.43	0.002
20:4n-6 ARA	153.0^b^	243.3^a^	234.7^a^	189.5^b^	253.3^a^	109.8^c^	13.62	<0.0001
20:5n-3 EPA	55.3^c^	53.5^c^	81.0^b^	85.1^b^	57.6^c^	125.0^a^	7.68	<0.0001
20:3n-6	26.2	22.7	24.0	27.4	30.8	25.5	2.53	0.303
20:4n-3	7.7	9.8	9.6	6.1	5.2	11.4	2.55	0.515
20:2n-6	3.0^b^	4.9^a^	5.9^a^	5.2^a^	6.0^a^	4.5^ab^	0.55	0.008
20:0	3.7^ab^	3.1^b^	5.2^a^	2.8^b^	3.2^b^	4.5^ab^	0.57	0.045
22:5n-6 DPA-6	5.4^b^	9.9^a^	8.7^a^	4.6^b^	7.4^ab^	4.7^b^	1.05	0.004
22:6n-3 DHA	124.6^bc^	108.7^c^	147.1^b^	152.5^b^	132.2^bc^	289.5^a^	12.19	<0.0001
22:5n-3 DPA-3	147.7^ab^	134.8^b^	161.9^ab^	169.6^a^	143.7^ab^	172.4^a^	9.52	0.050
22:0	6.1^ab^	5.2^b^	7.3^a^	6.1^ab^	6.0^ab^	5.9^ab^	0.55	0.032
23:0	21.2	16.4	19.4	18.5	16.6	18.1	1.67	0.355
24:0	12.6	11.0	13.9	12.2	12.0	13.0	1.20	0.642
Total FA	3093.0^b^	3373.0^ab^	3775.0^a^	3325.0^ab^	3296.0^ab^	2776.0^b^	188.40	0.024
∑SFA	1398.0^a^	1331.0^a^	1444.0^a^	1287.0^a^	1284.0^a^	1086.0^b^	63.30	0.008
∑MUFA	798.3^bc^	878.4^ab^	1018.9^a^	775.0^bc^	784.2^bc^	615.7^c^	66.48	0.007
∑PUFA	829.8^b^	1110.0^a^	1234.1^a^	1206.5^a^	1180.5^a^	1034.9^a^	64.34	0.001
∑n-3 LC-PUFA	335.3^cd^	306.7^d^	399.6^bc^	413.2^b^	338.7^cd^	598.2^a^	24.52	<0.0001
∑n-3 PUFA	396.9^d^	378.3^d^	503.9^bc^	542.9^b^	412.4^cd^	647.1^a^	32.47	<0.0001
∑n-6 PUFA	412.8^b^	716.4^a^	712.7^a^	653.6^a^	755.4^a^	382.5^b^	43.05	<0.0001
∑other FA	66.2^a^	53.5^ab^	70.8^a^	55.7^ab^	47.2^ab^	38.8^b^	7.49	0.045
n-6/n-3	1.0^c^	1.9^a^	1.4^b^	1.3^bc^	1.9^a^	0.6^d^	0.11	<0.0001

^*^Values within the same row bearing different superscripts differ (P < 0.05). All other abbreviations are as defined in Tables 1 and 2.

#### Fatty acid profiles in the heart

Table 4 depicts fatty acid contents (mg/100 g) of the heart. There was no difference in total FA, SFA and MUFA; similar to the same trend observed in the *longissimus dorsi* muscle. PUFA and 18:1n-7t in all supplemented treatments were significantly higher than in the control treatment. The control had the lowest contents of n-6 PUFA and LA. The RPO treatment had the highest n-3 LC-PUFA and n-3 PUFA, whilst the lowest contents of these FA were in the control treatment. FSO had the highest ALA content (51.3 mg/100 g). SO and rice bran infused pellets (RBO) treatments had the highest n-6/n-3 ratio and RPO had the lowest. Generally, the n-6/n-3 ration in all treatments ranged from 2.7 to 6.6.

**Table 4:** Fatty acid contents (mg/100 g) of the heart.

Fatty acid	Control	RBO	CO	FSO	SO	RPO	SEM	P
14:0	8.5	6.6	9.2	9.6	9.7	9.5	2.54	0.949
15:0	4.3	2.8	3.5	3.7	3.7	3.9	0.50	0.444
16:1n-9c	2.8	2.3	3.4	2.6	2.5	2.4	0.40	0.507
16:1n-7c	5.9^ab^	4.4^b^	5.6^ab^	5.3^ab^	4.9^ab^	7.7^a^	0.93	0.042
16:0	206.9	214.4	234.5	225.7	228.2	221.9	22.60	0.962
17:0	21.8^a^	14.5^b^	16.6^ab^	17.7^ab^	17.9^ab^	18.5^ab^	2.16	0.043
18:2n-6 LA	267.2^c^	489.2^a^	427.9^ab^	439.0^ab^	467.5^a^	365.9^b^	25.02	<0.0001
18:3n-3 ALA	23.2^c^	29.9^bc^	38.4^b^	51.3^a^	24.6^c^	27.5^bc^	3.73	<0.0001
18:1n-9	245.6	202.6	265.2	229.7	223.8	217.8	32.90	0.812
18:1n-7c	34.3^b^	38.8^b^	52.8^a^	37.3^b^	41.8^ab^	51.5^a^	4.03	0.011
18:1n-7t	21.0^c^	42.6^b^	44.3^b^	66.1^a^	53.2^ab^	51.6^ab^	6.42	0.001
18:0	309.0	312.2	327.2	332.8	342.1	271.5	35.17	0.769
20:4n-6 ARA	81.8^b^	128.6^a^	126.5^a^	101.7^ab^	135.0^a^	81.1^b^	10.82	0.002
20:5n-3 EPA	17.2^d^	25.0^bcd^	33.1^bc^	37.0^b^	21.2^cd^	65.6^a^	3.98	<0.0001
20:3n-6	9.6^b^	9.5^b^	9.9^b^	8.8^bc^	6.7^c^	12.5^a^	0.84	0.002
20:4n-3	2.4^bc^	1.3^c^	1.7^c^	1.5^c^	4.3^ab^	5.2^a^	0.78	0.004
20:2n-6	1.3^c^	1.9^bc^	2.2^ab^	1.9^bc^	2.5^ab^	2.7^a^	0.23	0.003
20:0	4.3	3.7	4.3	3.7	4.1	3.5	0.35	0.452
22:5n-6 DPA-6	1.0^b^	1.6^a^	1.3^ab^	1.0^b^	1.6^a^	1.5^ab^	0.17	0.042
22:6n-3 DHA	10.1^c^	14.8^bc^	17.2^b^	17.9^b^	15.8^bc^	39.8^a^	2.17	<0.0001
22:5n-3 DPA-3	25.3^c^	29.1^bc^	32.4^abc^	34.3^ab^	28.9^bc^	36.8^a^	2.39	0.024
22:0	6.0	6.1	5.8	5.4	5.8	5.2	0.31	0.336
23:0	11.6^a^	9.7^b^	9.0^bc^	9.3^b^	7.4^c^	8.9^bc^	0.59	0.001
24:0	6.4^ab^	6.7^a^	5.7^ab^	5.7^ab^	5.4^b^	5.9^ab^	0.39	0.045
Total FA	1611.0	1913.0	1992.0	1960.0	1961.0	1802.0	145.20	0.438
∑SFA	580.0	577.3	616.5	614.2	625.0	549.4	62.73	0.951
∑MUFA	360.8	344.0	431.7	406.1	384.0	386.8	46.16	0.803
∑PUFA	453.5^b^	745.7^a^	704.4^a^	705.4^a^	720.4^a^	646.4^a^	41.02	0.000
∑n-3 LC-PUFA	54.8^c^	70.2^bc^	84.4^b^	90.6^b^	70.1^bc^	147.5^a^	8.13	<0.0001
∑n-3 PUFA	78.2^d^	101.7^cd^	123.6^bc^	143.2^b^	95.7^cd^	175^a^	10.37	<0.0001
∑n-6 PUFA	363.9^c^	636.5^a^	572.6^a^	556.4^ab^	618.8^a^	467^b^	33.61	<0.0001
∑other FA	215.5	244.9	238.2	233.3	229.8	218.9	15.27	0.742
n-6/n-3	4.7^b^	6.3^a^	4.7^b^	4.1^b^	6.6^a^	2.7^c^	0.32	<0.0001

^*^Values within the same row bearing different superscripts differ (P < 0.05). All other abbreviations are as defined in Tables 1 and 2.

#### Fatty acid profiles in the kidney

Table 5 shows the fatty acid contents (mg/100 g) in the kidney. SFA contents did not differ among treatments. All supplemented treatments had significantly higher PUFA and LA contents than the control. RPO treatment had the highest n-3 LC-PUFA and n-3 PUFA contents. The highest total FA, n-6 PUFA and LA contents were in the SO treatment (2305.0; 752.6 and 375.8 mg/100 g, respectively). The observed trend of n-3 LC-PUFA content was similar to the pattern observed in the liver where only FSO treatment had greater n-3 LC-PUFA content than the control. The range in n-6/n-3 ratio in all treatments was from 1.3 to 4.0 in which the SO treatment had the highest ratio.

**Table 5:** Fatty acid contents (mg/100 g) of the kidney.

Fatty acid	Control	RBO	CO	FSO	SO	RPO	SEM	P
14:0	3.7^a^	3.3^ab^	3.7^a^	2.2^b^	3.0^ab^	3.2^ab^	0.42	0.046
15:0	4.9^a^	2.8^b^	3.9^ab^	3.4^b^	4.0^ab^	3.6^b^	0.40	0.018
16:1n-9c	3.1^b^	2.7^bc^	4.1^a^	2.0^c^	2.9^bc^	2.2^bc^	0.31	0.001
16:1n-7c	5.8^a^	3.8^bc^	5.0^ab^	3.2^c^	3.8^bc^	5.3^a^	0.42	0.001
16:0	270.1	285.3	309.1	264.5	341.0	276.6	25.50	0.293
17:0	24.0^a^	18.2^b^	19.7^ab^	18.8^b^	22.3^ab^	19.7^ab^	1.54	0.041
18:2n-6 LA	167.0^c^	282.0^b^	272.3^b^	293.6^b^	375.8^a^	238.4^b^	23.95	<0.0001
18:3n-3 ALA	24.6^a^	16.8^b^	27.7^a^	30.0^a^	18.2^b^	16.3^b^	1.85	<0.0001
18:1n-9	238.8^a^	198.1^ab^	244.2^a^	165.6^b^	215.2^ab^	159.6^b^	17.83	0.006
18:1n-7c	25.5^ab^	27.6^ab^	34.5^ab^	23.6^b^	35.6^a^	30.7^ab^	3.47	0.043
18:1n-7t	13.8^c^	26.8^bc^	30.4^ab^	45.1^a^	43.0^ab^	39.0^ab^	5.35	0.002
18:0	305.9^b^	319.5^ab^	349.1^ab^	331.8^ab^	399.1^a^	300.6^b^	28.37	0.038
20:4n-6 ARA	132.4^c^	255.1^b^	247.5^b^	208.6^bc^	334.7^a^	136.2^c^	27.11	<0.0001
20:5n-3 EPA	54.0^bc^	34.5^c^	59.9^bc^	71.0^b^	45.2^bc^	139.3^a^	11.20	<0.0001
20:3n-6	10.8	11.7	12.3	17.9	16.6	16.6	2.46	0.214
20:4n-3	7.7	5.1	3.0	2.6	4.5	4.8	1.86	0.467
20:2n-6	3.4^d^	8.8^ab^	6.2^c^	7.5^bc^	10.9^a^	5.5^cd^	0.78	<0.0001
20:0	6.5^b^	6.3^b^	6.5^b^	6.0^b^	8.1^a^	5.0^b^	0.52	0.011
22:5n-6 DPA-6	0.3^d^	2.6^a^	1.6^b^	0.7^cd^	2.5^a^	1.1^bc^	0.19	<0.0001
22:6n-3 DHA	27.7^c^	42.8^bc^	52.2^b^	51.2^b^	56.8^b^	97.3^a^	6.59	<0.0001
22:5n-3 DPA-3	32.2^c^	51.7^b^	63.9^ab^	70^a^	64.5^ab^	59^ab^	5.14	0.000
22:0	31.0^b^	35.4^ab^	36.3^ab^	34.4^b^	44.0^a^	29.8^b^	2.89	0.025
23:0	13.4	10.7	11.0	11.0	12.7	11.3	0.85	0.158
24:0	38.9^ab^	38.7^ab^	40.3^ab^	36.9^ab^	46.7^a^	35.5^b^	3.13	0.032
Total FA	1611.0^b^	1855.0^ab^	2015.0^ab^	1857.0^ab^	2305.0^a^	1772.0^b^	160.60	0.038
∑SFA	699.4	720.9	780.4	709.9	882.1	686.2	61.24	0.232
∑MUFA	358.8^ab^	330.7^ab^	400.9^a^	315.2^ab^	383.4^ab^	301.1^b^	28.89	0.031
∑PUFA	475.5^c^	730.7^b^	765.2^ab^	764.6^ab^	951.7^a^	721.8^b^	66.99	0.002
∑n-3 LC-PUFA	121.4^c^	134.0^bc^	179.1^bc^	194.7^b^	171.0^bc^	300.4^a^	20.81	<0.0001
∑n-3 PUFA	146.1^c^	150.8^c^	206.8^bc^	224.6^b^	189.2^bc^	316.8^a^	21.66	<0.0001
∑n-6 PUFA	317.7^d^	571.2^b^	548.5^bc^	533.8^bc^	752.6^a^	401.3^cd^	51.39	<0.0001
∑other FA	76.9^ab^	72.0^ab^	68.3^ab^	66.7^ab^	87.7^a^	62.7^b^	6.62	0.041
n-6/n-3	2.2^c^	3.8^a^	2.7^b^	2.4^bc^	4.0^a^	1.3^d^	0.17	<0.0001

^*^Values within the same row bearing different superscripts differ (P < 0.05). All other abbreviations are as defined in Tables 1 and 2.

## Discussion

Past studies had used raw oil or oilseeds as supplements in ruminant feeding trials. In order to minimise oxidative rancidity, improve stability and shelf life of both feeds and meat products, we chose to use oil-infused pellets as an innovative means of delivering n-3 LC-PUFA to lambs. Besides, available information on the effect of oil-infused pellets on the fatty acid profile of lamb is scanty in the published literature. Furthermore, the use of oil-infused pellets is less bulky than raw oil or oilseeds, hence our strategy minimises transportation and storage issues in commercial lamb feed production. The fatty acid profile of lucerne hay was similar to that reported by Nguyen *et al*.^10^. Many past studies had reported that flaxseed oil contained a high concentration of ALA^13–16^. Our study showed that FSO had the highest concentration of ALA among the supplemented treatments. The high concentration of LA in SO observed in this study was consistent with other previous studies^12,17^. The RPO treatment contained high concentrations of EPA, DPA and DHA because of its fish oil content.

In the *longissimus dorsi* muscle, the higher contents of n-3 LC-PUFA in the supplemented treatments can be explained by the higher levels of by-pass lipids and protection from excessive lipolysis and extensive biohydrogenation in the rumen^18,19^ as observed in previous studies^9,10,20^. On the other hand, it would seem that the levels and extent of biohydrogenation and conversion of stearate (18:0) into Oleate (18:1n-9) were similar, hence the observed result where no difference was found in the 18:1n-9 content of the *longissimus dorsi* muscle between the supplemented and control treatments. This observation could also be indicative of similar delta-nine desaturase enzyme activities among treatment groups since it is the primary enzyme responsible for the conversion of stearate to oleate in the biochemical pathway of *in vivo* lipid synthesis. One of the biohydrogenation intermediates of ALA and LA is 18:1n-7t^21^. The increased contents of ALA with flaxseed supplementation has also been reported in a previous study by Wachira *et al*.^22^ who reported that supplementation with flaxseed doubled the proportion (x100) of 18:3n-3 in the *longissimus dorsi* muscle from 1·4 to 3·1. Demirel *et al*.^23^ reported that feeding protected linseed resulted in 3.0-fold higher concentration of ALA in lamb muscles compared with Megalac (calcium soap of palm fatty acid distillate). The observed trend in our current study where the ALA content in the *longissimus dorsi* muscle of lambs in the FSO treatment was 1.9 times that of the control but similar EPA and DHA contents, could be due to the low elongase enzyme activity for the elongation of C18 to ≥C20 FA and subsequent desaturation of ALA into more potent n-3 LC-PUFA, and the capacity of muscle lipids to incorporate ALA into n-3 LC-PUFA^18^.

**Figure 1:**
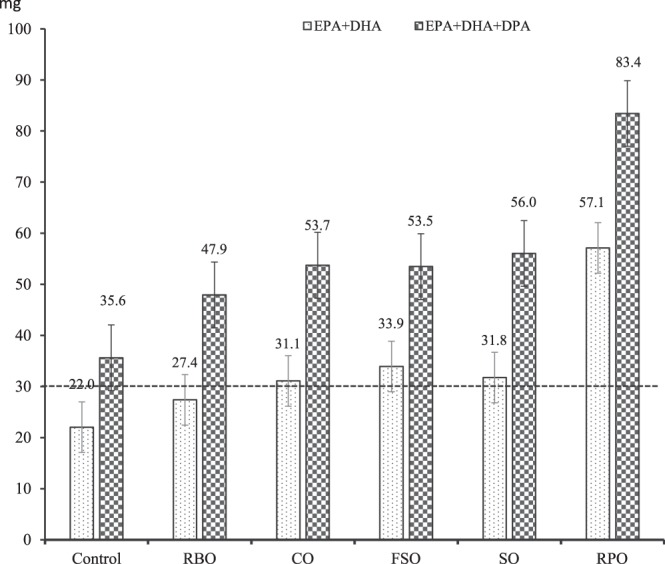
EPA + DHA and EPA + DHA + DPA contents per standard serve (135 g) in the *longissimus dorsi* muscle of prime lambs supplemented with diverse dietary PUFA sources (EPA: eicosapentaenoic acid; DPA: docosapentaenoic acid and DHA: docosahexaenoic acid; RBO, CO, RPO, FSO and SO were wheat-based pellets infused with 50 ml/kg DM of oil from rice bran, canola, rumen-protected, flaxseed and safflower sources, respectively).

In contrast, our finding that the RPO treatment had double EPA and triple DHA contents in *longissimus dorsi* muscle tissue in comparison with the control was in line with the results of Wachira *et al*.^22^ who reported that feeding fish oil increased the muscle proportion (x100) of EPA from 0·7 to 2·3 and DHA from 0·3 to 0·8. The greatest n-6 to n-3 ratio in SO treatment could be explained by high n-6 PUFA contents of safflower oil (57%) as shown in Table 1. According to Pannier, *et al*.^24^, a standard serve was 135 g of meat. Food Standards of Australia and New Zealand (FSANZ) regulate that a given food can be claimed as a ‘source’ of omega-3 fatty acids if the EPA + DHA contents of that food is greater than 30 mg per serve^25^. Lambs in the CO, FSO, SO and RPO treatments had EPA + DHA content in the *longissimus dorsi* muscle exceeding the 30 mg per serve cut-off point for “source” claim under the FSANZ guidelines^25^ (as depicted in Fig. 1) and the n-6/n-3 ratio within the desirable level of less than 4.0^26^. The DPA content is not currently considered as an omega-3 content claim under current regulations^27^; however, Australia and New Zealand have guidelines for total EPA + DHA + DPA consumption^28^. Byelashov, *et al*.^29^ revealed that DPA was related to various improvements in human health and served as a storage depot for EPA and DHA in the human body. Howe, *et al*.^27^ suggested that the importance of DPA in delivering the health benefits associated with n-3 LC-PUFA was equal to that of EPA and DHA. Therefore, if DPA was considered as an omega-3 content claim, a significant amount of n-3 LC-PUFA intake (~39%) would be added to lamb meat consumers as demonstrated in Fig. 1.

Edible by-products (organ meats) from cattle, pigs and lambs range from 20 to 30% of the animal’s liveweight^30^. The findings of Bester *et al*.^31^ showed that lamb organ meats are nutrient-dense animal source foods that provide protein, zinc and iron to meet the nutritional requirements of humans. Furthermore, the edible internal organs of prime lambs (heart, liver and kidney) are rich in essential fatty acids^32^. Edible internal organs are widely consumed in many countries as special ingredients in traditional foods such as kidney pie in the United Kingdom, cooked and diced delicacies in South America, sheep liver in Iran and paté in many countries^33^. Therefore, knowledge of the fatty acid profiles of lamb edible internal organs could contribute innovations in value-addition of edible meat by-products and generate extra income for lamb producers and slaughterhouses.

The liver is the first organ to receive and metabolise fatty acids after lipid digestion and absorption. As with previously reported studies^11,32^, individual fatty acid content was markedly high in the liver. Our observation of the lowest SFA and 18:0 contents in RPO treatment is supported by the work of Gallardo *et al*.^34^ who found that supplementation of ewes with 3% Ca soap FA in fish oil based diets dramatically reduced 18:0 content in milk. Shingfield *et al*.^35^ also observed that increased fish oil percentage in the diet linearly decreased 18:0 contents in the duodenum. The highest MUFA in CO (mainly 18:1n-9 contents) can be explained by the high MUFA (45.7%) input of CO into the diet. Similarly, the high LA and ALA contents (128.1 mg/100 g) in the liver of FSO fed lambs indicates that despite rumen biohydrogenation, a significant amount of ALA had escaped and was absorbed in the intestines and metabolised in the liver. Demirel *et al*.^23^ reported that feeding linseed more than doubled the concentration of ALA in the liver compared to Megalac diet. Out of all the PUFA of plant oil origin in this study, only FSO treatment had an n-3 LC-PUFA content that was significantly higher than the control. This could be explained in part, by the high ALA content in FSO being converted to n-3 LC-PUFA. Our result was in line with the findings of Alhazzaa *et al*.^36^ who reported that the gradual increase in ALA concentration led to a proportional increase in EPA in hepatocytes. The liver EPA and DHA contents of PUFA fed lambs ranged from 162.2 to 414.0 mg/100 g which was similar to EPA and DHA contents of wild Australian seafoods such as fish (containing 350 mg/150 g (~233 mg/100 g), shellfish (containing 225 mg/150 g (~150 mg/100 g) (see the review by Nichols *et al*.^37^). Furthermore, the n-6/n-3 ratio in the liver is within desirable level^26^. Therefore, the n-3 LC-PUFA content in the liver of PUFA fed lambs can be labelled as reaching the claimable ‘good source’ based on EPA + DHA threshold of the Food Standards of Australia and New Zealand guidelines^25^. Therefore, if lamb consumers consider the liver as their dietary protein source, they would obtain the associated dietary health benefits. In the heart, Kashani *et al*.^38^ reported that supplementation of lambs with Spirulina at the medium-level increased both n-3 and n-6 PUFA compositions. The results from our study showed that total PUFA, n-3 LC-PUFA and n-6 PUFA contents of heart were all enhanced by supplementation with various PUFA enriched pellets. Furthermore, n-3 LC-PUFA contents in the heart of supplemented lambs ranged from 70.1 to 147.5 mg/100 g; indicating that the heart could be a ‘good source’ of n-3 LC-PUFA^25^. However, the n-6/n-3 ratio of the heart in all lambs with the exception of RPO, was much higher than the minimum desirable level of 4.0^26^. The kidney samples in the present study had high contents of key health-benefitting n-3 LC-PUFA in agreement with the findings of Malau-Aduli *et al*.^32^. Specifically, our current findings showed that the contents of n-3 LC-PUFA ranged from 121.4 to 300.4 mg/100 g which is equal to the n-3 LC-PUFA content in Australian wild fish or shellfish (see details in the review by Nichols *et al*.^37^). The result of the present study demonstrated that supplementation of lambs with PUFA enriched pellets substantially increased kidney n-3 LC-PUFA contents. Nguyen *et al*.^11^ also reported that supplementation of lambs with canola and flaxseed oils at 5% enhanced n-3 LC-PUFA contents of the kidney.

## Conclusion

Supplementation of lambs with PUFA enhanced pellets in a feedlot system is needed to boost the contents of n-3 LC-PUFA in the *longissimus dorsi* muscle. Lambs in CO, FSO, SO and RPO treatments had contents of EPA + DHA in *longissimus dorsi* muscle that were over the claimable ‘source’ level of omega-3 as stipulated by the Foods Standards of Australia and New Zealand guidelines. Supplementation of lambs with oils of plant origin including canola, rice bran, flaxseed and safflower had similar effects on the contents of n-3 LC-PUFA in lamb *longissimus dorsi* muscle. FSO had the highest contents of ALA in the muscle, liver and heart tissues. Liver and kidney of PUFA fed lambs can be utilised as omega-3 rich foods on the basis of their high n-3 LC-PUFA contents and desirable n-3/n-6 ratios. In line with the tested hypothesis in this study, it is proven that canola, flaxseed, safflower and rumen protected oil infused pellets can be used in feedlot systems for producing healthy lamb meat as a source of omega-3 food. The cost-efficacy of supplementing lambs with these oil-infused pellets has been presented in detail in a separate stand-alone manuscript (currently under review) and details will not be duplicated herein. Suffice it to say that the feed cost per unit gain in Australian dollars/kg (AU$/kg) for RBO and CO supplements were 3.0 AU$/kg each, 4.1, 4.2 and 6.3 AU$/kg for RPO, FO and SO supplements, respectively.

## Materials and Methods

This study was carried out at the Tasmanian Institute of Agriculture’s Cressy Research and Demonstration Station, Burlington Road, Cressy, Tasmania, Australia (41° 43′S, 147° 03′E) from April to June, 2016. The use of animals and procedures performed in this study were all approved by the University of Tasmania Animal Ethics Committee (Permit No A0015657).

### Animals, diets and experimental design

Seventy-two White Suffolk x Corriedale first-cross prime lambs with an average liveweight (LWT) of 35.7 ± 0.9 kg weaned at 6 months were randomly allocated into six treatments: (1) Control: lucerne hay only; wheat-based pellet infused with 50 ml/kg dry matter (DM) of oil from (2) rice bran (RBO); (3) canola (CO); (4) rumen-protected (RPO), (5) flaxseed (FSO) and (6) safflower (SO) sources in a completely randomized design balanced by equal number of ewe and wether lambs. The animals were kept in individual pens and had *ad libitum* access to clean fresh water and lucerne hay throughout the duration of the feeding trial. The lambs were sheltered in a roofed experimental shed with adequate ventilation and individual lamb pen dimension was 6 m^2^ (1.2 m in width and 5 m in length). The average minimum and maximum temperatures during the experimental period were 6.5 °C and 19.7 °C in April and 3.4 °C and 12.6 °C in June, respectively. Average rainfall figures during the experimental period were 21.4 mm and 147 mm in April and June, respectively. Each lamb in the supplemented treatments was fed 1 kg pellets/day. The feeding trial lasted 10 weeks including a 3-week adaptation period and followed by 7 weeks of full supplementation. Freshly offered feed was given every day at 9.00 h after residual feed had been collected and weighed. The level of refusal of the concentrate supplement ranged from 0.14–0.18 kg DM/head/day. The chemical composition of the experimental and basal diets is presented in Table 6.

**Table 6:** Proximate analysis of the experimental and basal diets.

Chemical composition (% DM)	Lucerne hay	RBO	CO	FSO	SO	RPO
DM	86.8	89.9	91.0	90.7	89.9	89.7
Protein	17.1	14.8	14.0	14.6	14.5	15.6
ADF	36.9	7.5	9.4	10.4	10.0	8.2
NDF	47.2	19.0	19.1	22.2	21.1	20.4
EE	1.5	5.5	5.6	5.6	5.5	5.1
ASH	8.4	6.7	6.2	8.2	8.2	6.5
%TDN	60.2	83.1	81.6	80.8	81.1	82.5
DE (Mcal/kg)	2.65	3.65	3.59	3.56	3.57	3.63
ME (MJ/kg)	9.08	12.54	12.32	12.20	12.25	12.46

Dry matter (DM), Neutral detergent fibre (NDF), Acid detergent fibre (ADF), Ether extract (EE) and crude protein (CP), Total digestible nutrients (%TDN), Metabolisable energy (ME). Total digestible nutrients (%TDN) were calculated as TDN (% of DM) = 82.38 − (0.7515 × ADF [% of DM]). Metabolisable energy (ME) was calculated by converting %TDN to digestible energy (DE [Mcal/kg] = %TDN × 0.01 × 4.4) which was converted as ME = (DE (Mcal/kg) × 0.82) × 4.185. All other abbreviations are as defined in Table 1.

### Procedures of feed sample and chemical composition determination

Every week, feed samples were taken from each pellet bag and bale of lucerne hay and bulked. The bulked samples were stored at −20 °C until the end of the feeding trial. The feed samples were dried at 60 °C over 72 h, ground to pass through a 1 mm sieve using a Laboratory Mill (Thomas Model 4 Wiley® Mill; Thomas Scientific) and analysed using the standard laboratory analytical methods of AOAC^39^ for DM and ash. Neutral Detergent (NDF) and Acid Detergent (ADF) fibre contents were determined using an Ankom Fibre Analyzer (ANKOM2000; ANKOM Technology, USA). Nitrogen content was quantified using a Thermo Finnigan EA 1112 Series Flash Elemental Analyzer and the values multiplied by 6.25 to give the estimated crude protein (CP) percentage. Ether extract (EE) was analysed using an ANKOM^XT15^ fat/oil extractor (ANKOM Technology, USA).

### Slaughter protocol and fatty acid analysis

All animals were fasted overnight before transporting them to a near-by commercial abattoir (Tasmanian Quality Meats, Cressy, Tasmania) adjacent to the experimental site in strict compliance with the slaughter procedures prescribed by the Meat Standards of Australia guidelines^40^. Heart, kidney and liver samples were taken immediately after evisceration. All samples were vacuum-sealed, code-labelled and stored at −20 °C until fatty acid analysis. Thereafter, the carcasses were chilled for 24 h at 4 °C and *longissimus dorsi* muscle sampled at the 12/13^th^ rib of each carcass as a commercial loin chop (approximately 200 g) for FA analysis.

FA analyses of meat, organs and feed samples were conducted at the Commonwealth Scientific and Industrial Research Organization (CSIRO) Food Nutrition & Bio-based Products, Oceans & Atmosphere Laboratory in Hobart, Tasmania, Australia. The procedure of FA analysis was described in detail previously by Malau-Aduli, *et al*.^32^. In short, total lipids in 1 gram of un-homogenised and wet liver, kidney, heart and muscle tissue and feed samples were extracted overnight using a modified Bligh and Dyer^41^. The first step of the process was a single-phase overnight extraction using CHCl_3_:MeOH:H_2_O (1:2:0.8 v/v). The second step involved phase separation with the addition of CHCl_3_:saline Milli-Q H_2_O (1:1 v/v) and followed by rotary evaporation of the lower chloroform phase at 40 °C to obtain total lipids. An aliquot from each total lipid extract was used in transmethylation process with MeOH:CHCl_3_:HCl (10:1:1 v/v) for 2 h at 80 °C and then fatty acid methyl esters (FAME) were extracted three times using hexane:CHCl_3_ (4:1 v/v). A known concentration of an internal standard (19:0) was added in 1500 µL vial containing the extracted FAME. The FAME was analysed on a 7890B gas chromatograph (Agilent Technologies, Palo Alto, CA, USA) equipped with an Equity^TM^-1 fused 15 m silica capillary column with 0.1 mm internal diameter and 0.1-μm film thickness (Supelco, Bellefonte, PA, USA), a flame ionisation detector, a split/splitless injector and an Agilent Technologies 7683 B Series autosampler. The gas chromatograph conditions were: splitless mode injection; carrier gas He; initial oven temperature 120 °C and then increased to 270 °C at 10 °C/min and to 310 °C at 5 °C/min. The Agilent Technologies ChemStation software (Palo Alto, California USA) was used to quantify fatty acid peaks. The fatty acid identities were confirmed by gas chromatograph-mass spectrometric (GC/MS) analysis using a Finnigan Thermoquest GCQ GC/MS fitted with an on-column injector and Thermoquest Xcalibur software (Austin, Texas USA). The gas chromatograph (GC) was equipped with an HP-5 cross-linked methyl silicone-fused silica capillary column (50 m × 0.32 mm internal diameter) which was of similar polarity to the column described above. The carrier gas was helium and GC conditions were according to the description of Miller, *et al*.^42^. FA percentages were computed as follows: FA% = (individual fatty acid area) ∗ (100)/(sum total area of fatty acids). FA contents were calculated as follows: FA mg/100 g = (Total lipid) ∗ (LCF [0.916]) ∗ ([%FA]/100) ∗ 1000^43^, where 0.916 was the lipid conversion factor cited by Clayton^44^.

### Statistical analysis

Fatty acid data were initially transformed into fatty acid contents (mg/ 100 g). These data were then analysed using the General Linear Model procedure (PROC GLM) of Statistical Analysis System^45^. The fixed effects of treatment and gender and their second order interaction effects on fatty acid contents were tested. The initial full statistical model used for the analysis was: Y = μ + O_i_ + G_j_ + (OG)_ij_ + (OG)^2^_ij_ + (OG)^3^_ij_ + e_ijk_ where Y = dependent variable, μ = overall mean, O_i_ = oil supplementation treatment, G_j_ = gender, brackets and superscripts represent linear and cubic second-order interactions and e_ijk_ = residual error. Linear and cubic orthogonal contrasts indicated that gender was not a significant factor, hence it was removed from the final model that assessed the impact of treatment only. Duncan’s multiple range tests were used to determine the differences amongst treatments at a minimum threshold of p < 0.05 level.

## Data Availability

The datasets generated during and/or analysed during the current study are available from the corresponding author on reasonable request.
